# Restoration of miR-193a-5p and miR-146 a-5p Expression Induces G1 Arrest in Colorectal Cancer through Targeting of MDM2/p53

**DOI:** 10.15171/apb.2020.017

**Published:** 2019-12-11

**Authors:** Saeed Noorolyai, Elham Baghbani, Leili Aghebati Maleki, Amir Baghbanzadeh Kojabad, Dariush Shanehbansdi, Vahid Khaze Shahgoli, Ahad Mokhtarzadeh, Behzad Baradaran

**Affiliations:** ^1^Immunology Research Center, Tabriz University of Medical Sciences, Tabriz, Iran.; ^2^Student Research Committee, Tabriz University of Medical Sciences, Tabriz, Iran. Introduction

**Keywords:** Colorectal cancer, miRNA-193a-5p, miRNA-146a-5p, MDM2/p53, Cell cycle, Restoration

## Abstract

***Purpose:*** Colorectal cancer (CRC) remains a universal and lethal cancer owing to metastatic and relapsing disease. Currently, the role of microRNAs has been checked in tumorigeneses. Numerous studies have revealed that between the tumor suppressor miRNAs, the reduced expression of miR-146a-5p and -193a-5p in several cancers including CRC tissues are related with tumor progression and poor prognosis of patients. The purpose of this study is to examine the role of miR-146 a-5p and -193 a-5p in CRC cell cycle progression.

***Methods:*** The miR-193a-5p and -146 a-5p mimics were transfected into HT-29 CRC cells via jetPEI transfection reagent and their impact was assessed on p53, cyclin B, and NF-kB gene expression. The inhibitory effect of these miRNAs on cell cycle was assessed by flow cytometry. The consequence of miR-193a-5p and miR-146 a-5p on the protein expression level of Murine double minute 2 (MDM2) was assessed by western blotting.

***Results:*** miR193a-5p and -146a-5p regulated the expression of MDM2 protein and p53, cyclin B, and NF-kB gene expression in CRC cells. Treatment of HT-29 cells with miRNA-146a-5p and -193a-5p induced G1 cell cycle arrest.

***Conclusion:*** The findings of our study suggest that miR146a-5p and -193a-5p may act as a potential tumor suppressor by their influence on cell cycle progression in CRC cells. Thus, miRNA-146a-5p and -193a-5p restoration may be recommended as a potential therapeutic goal in the treatment of CRC patients.

## Introduction

Colorectal cancer (CRC) is the third most prevalent cancer and second largest reason of cancer death in Europe and North America.^1^ Its general frequency is 5% and the 5-year survival rate varies from 40 to 60%.^2^ Nevertheless, the accurate genetic and epigenetic aberrations in colon cancer development have not been comprehended entirely and further studies need to be carried out in this regard. MicroRNAs (miRNAs, miR) are endogenously stated small noncoding RNAs that prevent gene expression by base-pairing with complementary sequences of the 3´-untranslated region (3’-UTR) of messenger RNAs.^3^ Owing to the extensive regulator of gene expression, miRNAs take part in critical roles in several biological progressions, as well as metabolism, cell growth, transformation, and apoptosis.^4^ Dysregulations of miRNAs might be consequently found with cancer progression and carcinogenesis. Investigations have discovered that multiple miRNAs are upregulated or downregulated in CRC and might contribute to the pathogenesis of CRC via directing the expression of crucial signaling molecules.^5^ Growing proofs have established that miRNAs function as tumor suppressors or oncogenes, proposing their significant probability as novel therapeutic goals.^6^ Multiple studies have revealed that between the tumor suppressor miRNAs, miR-146 a-5p and -193 a-5p expression in multiple cancers such as CRC are linked with tumor progression, chemotherapy resistance, metastasis, and poor prognosis of patients.^7,8^ Considering essential molecular apparatuses in CRC development and progression gives new prophecies in emerging unique factors for CRC cures.^9^ However, insufficient clues exist establishing the dependency among miRNAs and target genes in CRC and to disband their roles in tumorigenesis.^10^

MDM2–p53 pathway is regularly affected in CRC and is strongly related with poor prognosis.^11^ Murine double minute 2 (MDM2) was duplicated from 3T3-DM, the transformed mouse cell line. MDM2 is the negative modulator of p53 gene and performs through impeding p53 transcriptional activity and transposition from the nucleus to the cytoplasm. The tumor suppressor protein p53 performs through directing downstream apparatus in cell cycle that clues to cell growth/postponement via apoptosis, DNA repair, and several other apparatuses.^12^ Moreover, p53 is a crucial controller of cell cycle checkpoints and apoptosis, directing the transcription of genes associated with over-proliferation, together with p21 and BAX.^13^ Adjustment of MDM2 gene expression is related with some transcription factors as well as NF-kB; furthermore, the MDM2 gene has been stated to have NF-kB binding sites.^14^

The aim of this study was to restore and increase the expression levels of miRNA-193a-5p and -146a-5p in the CRC cell line using microRNA replacement therapy. It also attempted to explore the effects of this miRNA replacement in multiple molecular and cellular aspects of CRC cells such as cell cycle controlling.

## Materials and Methods

### Cell culture

HT-29 cell line was purchased from Pasture Institute (Tehran, Iran). This cell line was cultured in RPMI‐1640 medium with 100 µg/mL streptomycin and 100 IU/mL penicillin (Gibco, Maryland) and completed with 10% fetal bovine serum (FBS; Gibco Laboratories, Grand Island, NY). Cells were retained in an incubator with 5% CO2 and 95% moistened atmosphere at 37°C.

### Transfection of miRNAs

For this aim 2 × 10^5^ HT-29 cells were cultured in a 6-well-plate with RPMI-1640 medium. After 24 h, the medium was changed with FBS and antibiotics-free Opti-MEM (Gibco Life Technologies, Gaithersburg, MD). The microRNA-146a-5p mimic and microRNA-193a-5p mimic with 100 pmol concentration were transfected to HT-29 cells separately and simultaneously using jetPEI in vitro transfection reagent (Polyplus, Illkirch, France) and conforming to the manufacturer’s guidelines.

The N/P ratio is a measure of the ionic balance of the complexes. It refers to the number of nitrogen residues of jetPEI™ per miRNA phosphate. Approximately one in three nitrogen atom of PEI is a cation, so electroneutrality of jetPEI™/miRNA complexes is reached for N/P = 2-3.

In practice, the best transfection results are obtained for N/P = 5 - 10. jetPEI™ is supplied as a 7.5 mM solution (expressed in nitrogen residues) and 1 µg of miRNA contains 3 nmoles of anionic phosphate.

### RNA isolation and quantitative real-time PCR (qRT-PCR)

Whole RNA was extracted from the cells using a RiboEX reagent (GeneAll; GeneAll Biotechnology, Seoul, Korea). The quality and the amount of extracted RNAs were determined with NanoDrop spectrophotometer (Thermo Fisher Scientific Life Sciences, USA). The mRNAs were reverse transcribed to cDNA using cDNA Synthesis Kit from Biofact Company (Daejeon, Korea) and following the manufacturer’s protocols.

The qRT‐PCR test was conducted using the LightCycler 96 (Roche Diagnostics, Mannheim, Germany) and SYBR green‐master mix (Biofact, Daejeon, Korea).

The primer sequences have been presented in Table 1. The assessment of p53, cyclin B, and NF-kB was carried out by 10 minutes of initial denaturation at 94°C followed by 45 cycles of 94°C for 10 seconds and 60°C for 60 seconds. Finally, using 2^-ΔΔCt^ cycle threshold method, the relative expression levels of genes were standardized with internal control U6 snRNA and GAPDH for miRNA and mRNA, respectively.

**Table 1:** The primers sequences

**Name**	**Forward and reverse**	**Sequences**
NF-kB	F	5′-GCTACACAGGACCAGGGACAGT-3’
	R	5′-AGCTCAGCCTCATAGAAGCCATC-3’
Cyclin B	F	5ˊ-TTGGTGTCACTGCCATGTTT-3ˊ
	R	5ˊ-GATGCTCTCCGAAGGAAGTG-3ˊ
p53	F	5ˊ-AAAGTCTAGAGCCACCGTCC-3ˊ
	R	5ˊ-AATCCAGGGAAGCGTGTCA-3ˊ
GAPDH	F	5ˊ-CAAGATCATCAGCAATGCCT-3ˊ
	R	5ˊ-GCCATCACGCCACAGTTTCC -3ˊ

Table 1 illustrates the primer sequences.

### Cell cycle analysis using DAPI and flow cytometry

Cells were harvested 48 hours after transfection and washed with clod PBS. Cell pellets were fixed with 70% ethanol at -20°C overnight. Subsequently, cells were washed with cold PBS and resuspended in 500 μL PBS. Then, 5 μL RNase A was added (10 mg/mL, Pishgam Biotech Co, Iran) and incubated for 30 minutes at 37°C. Next, it was resuspended in 500 μL PBS, and 1 μL DAPI (5 mg/mL, Sigma) and 1 μL Triton-x100 (ACROS Organics, USA) were added and protected from light. Samples were analyzed through flow cytometry (MacsQuant Analyser 10, Miltenyi Biotech, Germany).

### Western blotting

The total protein of whole cells was extracted using RIPA lysis buffer (Santa Cruz Biotechnology) and following the manufacturer’s procedure. Fifty micrograms of extracted protein was added on 4% stacking and 12.5% running gel on SDS-PAGE electrophoresis; then, protein bands were blotted into the polyvinylidene difluoride (PVDF) (Roche Diagnostics GmbH, Germany) membrane through semidry immunoblotting. The membrane was blocked with 0.5% Tween and then incubated with MDM-2 and β-actin (as a housekeeping protein) monoclonal antibodies with 1: 1000 concentrations overnight at 4°C (Santa Cruz Biotechnology, USA). Next, the membrane was incubated with rabbit anti-goat secondary antibody for MDM2 and rabbit anti-mouse antibody for β-actin, conjugated with horseradish peroxidase (1:2000; diluted in PBS) for 1 hour at room temperature. The protein bands were visualized using the electrochemiluminescence (ECL) kit (Roche Diagnostics, Germany) and western blot imaging system (Sabz Bimedicals Co., Iran). Finally, the amount of proteins was examined using the ImageJ software (National Institutes of Health, Bethesda, MD) and standardized with the β-actin.

## Results and Discussion

### The miR-193a-5p and -146a-5p replacement altered the expression of p53, cyclin B, and NF-kB in HT-29 cell line

**Figure 1:**
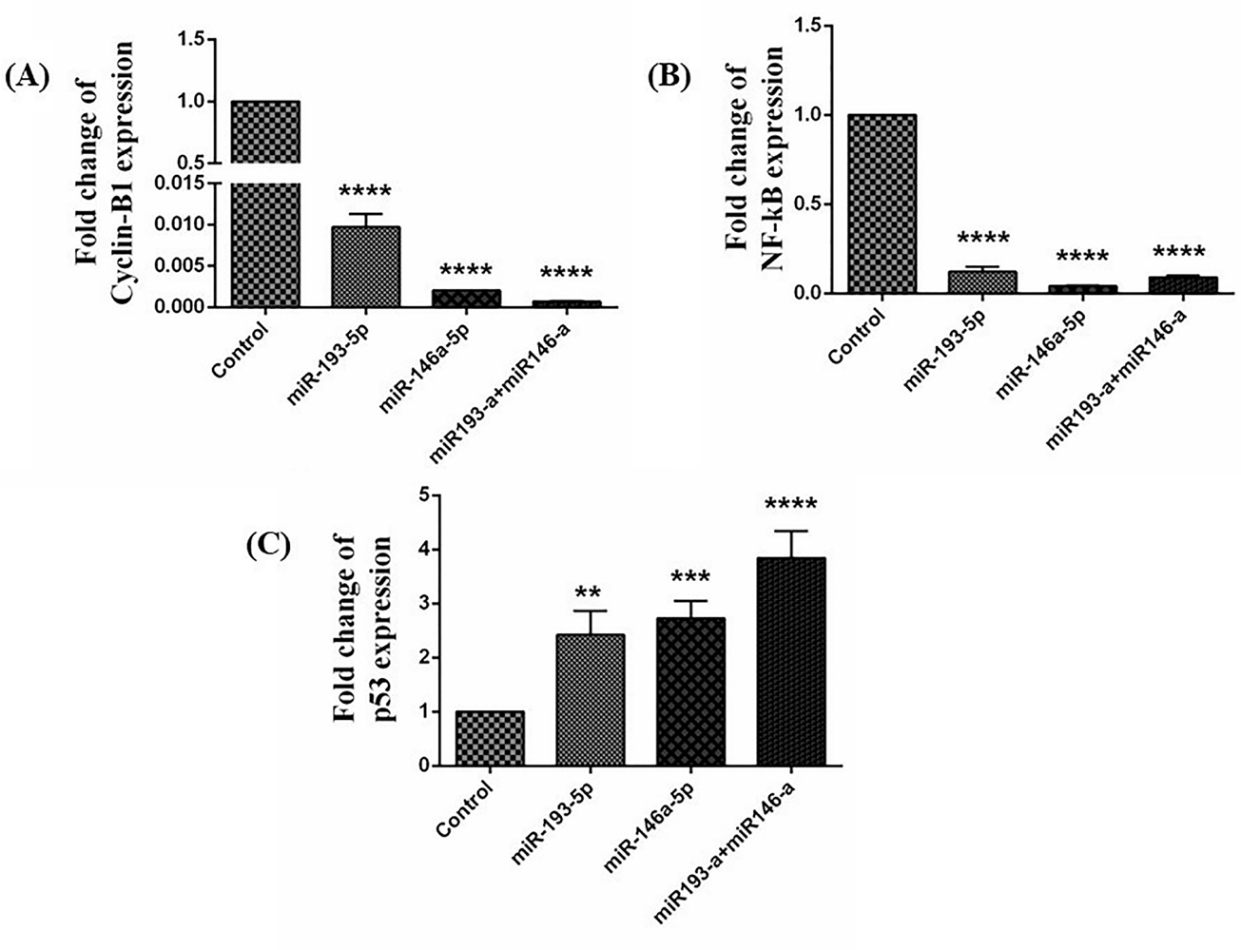
**The miR-193a-5p and -146a-5p restoration altered the expression of p53, cyclin B, and NF-kB in HT-29 cell line.** The outcomes of transfection of miRNA-146a-5p and -193a-5p alone and in combination with each other on the expression of (A)cyclin, (B) NF-kB, and (C) p53 in comparison with the control group (**P< 0.01, ***P< 0.001, **** *P*< 0.0001).

According to the results of qRT-PCR, while co-transfection and single-transfection with miRNA-146a-5p and -193a-5p significantly decreased the expression levels of cyclin B (Figure 1A) and NF-kB (Figure 1B), they increased the expression level of p53 (Figure 1C).

The association between NF-κB and p53 and its importance in the expansion and progression of cancer are fine recognized. NF-κB is crucial for p53-mediated cell death.^15^ In our study, the expression level of NF-κB was significantly reduced, and the expression of p53 was increased after miRNA-146a-5p and -193a-5p replacement. Along with these findings, we showed that transfection of miR-193a-5p and miR-146a-5p individually and simultaneously in HT-29 cell line increased the expression level of p53.

### Co-treatment with miR-146a-5p and -193a-5p significantly suppressed MDM2 protein in HT-29 cells

**Figure 2:**
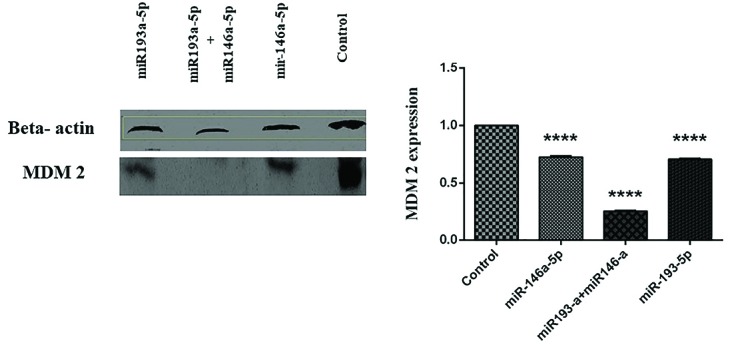
** The miR193a-5p and -146a-5p regulated the expression of MDM2 in CRC cell line**. The immunoblotting of MDM2 protein was evaluated in untransfected (control) and miR193a-5p and miR146a-5p separately and simultaneously transfected groups (The data represent mean ± SD, ****P < 0.0001).

To define the consequences of miRNA-146a-5p and -193a-5p restorationon the activity of MDM2, we considered mimic replacement incolorectal cell line. The replacement of these miRNAsalone and in combination with each other affected the activity of MDM2. The miRNA-146a-5p and miRNA-193a-5p individually led to the reduction of MDM2 protein compared to control group. This evidence is particularly more significant in a group of cells transfected simultaneously with both of these miRNAs (The data represent mean ± SD. n = 3, ****P < 0.0001, Figure 2).

In CRC, the overexpression of MDM2 is an early occurrence in the tumor progression. The MDM2 protein controls the action of p53 through developing a protein compound with p53, which could result in the beginning of p53 tumor suppressor role.^16^ Several studies and evidences have showed that MDM2 might be controlled by multiple cellular pathways, for example those related with miRNAs.^17,18^ Recently, abnormal miRNA expression profiles in multiple cancers including CRC have been reported by numerous studies.^19^ According to several studies, the expression levels of miR-193a-5p and miR-146a-5p are substantially reduced in CRC^8,20^; these findings were confirmed by the results of our study. In the present study, we observed that replacement of miR-193a-5p and miR-146a-5p individually and simultaneously in HT-29 CRC cell line reduced the ratio of MDM2 protein.

### The miR-193a-5p and -146a-5p replacement induced G1 cell cycle arrest

**Figure 3:**
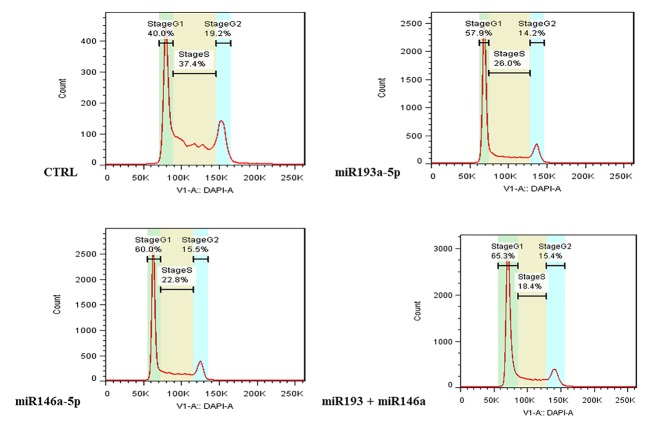
** The effect of miR-193a-5p and -146a-5p restoration on cell cycle progression of CRC cells.** The miRNA-193a-5p and -146a-5p alone and in combination with each other caused G1 arrest in HT-29 cells.

We examined cell cycle progression in different groups of transfected HT-29 cells. As shown in Figure 3, in the groups transfected with miRNA-193a-5p and -146a-5p mimic, as well as transfected cells with both miRNAs simultaneously, G1 arrest occurred in cell cycle progression.

According to these findings, miR-193a-5p and miR-146a-5p inhibit cell growth and induce p53 dependent cell death. Also, we demonstrated that replacement of these miRNAs induced G1 phase arrest in HT-29 cells. Similar to our research, Liu et al showed that transfection of miRNA-193 into DU-145 cells of prostate cancer caused cell cycle arrest in the G1 phase.^21^ Also Li et al established that replacement of miRNA-146 in lung cancer cell lines caused cell cycle arrest in the phases G1 and G0.^22^ The p53 is a tumor suppressor gene that is controlled by miRNAs. Several oncogenic miRNAs straightly target p53, while specified tumor-suppressive miRNAs target negative regulators of p53, including the MDM2 and E3 ligase.^23,24^ Cross talk among p53 and miRNAs is multifaceted and numerous miRNAs control the expression and function of p53 such as miR146a-5p and miR193a-5p.^25,26^ Li et al reported that miR-193a directly targets MDM2, and also miR-193a persuades G1 arrest and apoptosis and returns leukemic cell differentiation; though, it is currently mysterious as this happens through stimulation of p53.^26,27^

## Conclusion

In summary, our results displayed that miR146a-5p and miR193a-5p may affect MDM2 and p53 regulatory complex that controls CRC cells growth and expansion. Also, the results of current study suggest that miR146a-5p and miR193a-5p might function as a probable tumor suppressor displayed by their inhibition of cell cycle progress and elevation of apoptosis in CRC. Beside the assumption that miR146a-5p and miR193a-5p may have several target genes, these different target genes might apply numerous functions of these miRNAs in these biological procedures, enhancing vital queries for upcoming investigations.

## Ethical Issues

Not applicable.

## Conflict of Interest

The authors declare that there are no conflicts of interest.

## Acknowledgments

This study was supported by Immunology Research Center of Tabriz University of Medical Sciences, Tabriz, Iran.
